# Editorial: Role of long non-coding RNA and Circular RNA in bone metabolism and their role as circulating biomarkers for bone diseases

**DOI:** 10.3389/fendo.2023.1321962

**Published:** 2023-11-14

**Authors:** Martina Faraldi, Marta Gomarasca, Chong Yin, Giovanni Lombardi

**Affiliations:** ^1^ Laboratory of Experimental Biochemistry & Molecular Biology, IRCCS Istituto Ortopedico Galeazzi, Milano, Italy; ^2^ Department of Clinical Laboratory, Ministry of Science and Technology, Affiliated Hospital of North Sichuan Medical College, Nanchong, China; ^3^ Department of Oncology, Ministry of Science and Technology, Affiliated Hospital of North Sichuan Medical College, Nanchong, China; ^4^ Department of Rehabilitation Medicine, Ministry of Science and Technology, Affiliated Hospital of North Sichuan Medical College, Nanchong, China; ^5^ Department of Laboratory Medicine, Translational Medicine Research Center, North Sichuan Medical College, Nanchong, China; ^6^ Lab for Bone Metabolism, Xi’an Key Laboratory of Special Medicine and Health Engineering, Xi’an, Shaanxi, China; ^7^ Key Lab for Space Biosciences and Biotechnology, Research Center for Special Medicine and Health Systems Engineering, NPU-UAB Joint Laboratory for Bone Metabolism, School of Life Sciences, Northwestern Polytechnical University, Xi’an, Shaanxi, China; ^8^ Department of Athletics, Strength and Conditioning, Poznań University of Physical Education, Poznań, Poland

**Keywords:** bone biomarkers, long non coding RNA (lncRNA), bone metabolism, bone homeostasis, bone disorders

Long non-coding (lncRNAs) and circular RNAs (circRNAs) are two classes of non-coding RNA species (ncRNAs) whose length ranges from 200 to tens of thousands nucleotides and are involved in the regulation of gene expression (1).

Being central players in gene regulatory networks, both lncRNAs and circRNAs often interact with other biomolecules, such as coding and other non-coding RNAs, DNA, and proteins, to carry out their biological activity. Considering their regulatory functions, these ncRNAs are involved in multiple biological processes and in the regulation of physiological and developmental processes. However, they are key players in the etiology of several disease states and their deregulation or mutation are associated with different diseases.

lncRNAs and circRNAs are implicated in the regulation of bone turnover, a highly dynamic process orchestrated by osteoclasts, osteoblasts and osteocytes and also involve recruitment and differentiation of their progenitor cells (2). Both lncRNAs and circRNAs regulate the expression of genes involved in bone cell activity and homeostasis, through the construction of a lncRNA or circRNA/miRNA/mRNA network (3). Therefore, any alteration of these molecules or deregulation in these networks would potentially represent a crucial factor in the onset of bone disease.

This Research Topic is aimed at collecting the newest research insights about lncRNAs and circRNAs biological role in bone disorders.

In the study by An et al. lncRNA alterations have been investigated in the context of osteoporosis (OP). OP is characterized by the decrease of bone mineral density, due to imbalanced activities of osteoblasts and osteoclasts. As detailed by An F. et al, a leading cause of this imbalance is represented by the impaired osteogenic differentiation of bone marrow stromal cells (BMSCs), that during aging or under pathological stimuli, preferentially differentiate into adipocytes. This results in bone marrow adiposity, impaired osteoblastogenesis and, therefore, impaired bone formation. As summarized by An F. et al, BMSC osteogenic vs. adipogenic differentiation is regulated by a lncRNA/miRNA network that targets key transcription factors for osteoblast differentiation, e.g., Runx2, bone morphogenic proteins (BMPs), as well as key factors within the bone homeostasis signalling pathways (Wnt/β-catenin, TGFβ1/Smad2/3, PI3K/AKT).

Among the relevant lncRNAs in bone biology, MALAT1 is one of the most studied. As detailed by Zhang et al., MALAT1 can been considered as a biomarker for OP since its circulating levels are reduced in OP patients, compared to healthy subjects. Moreover, it was observed as involved into osteogenic differentiation of BMSCs, by inhibiting different miRNAs and promoting the expression of osteogenic transcription factors. However, MALAT1 has been also implied in the development of other bone and cartilage diseases: specifically, its decreased expression in chondrocytes is associated with osteoarthritis, while its expression is enhanced in intervertebral disc degeneration, rheumatoid arthritis, idiopathic arthritis, ankylosing spondylitis, and gouty arthritis.

lncRNAs associated with the regulatory process of bone homeostasis may provide ideal candidates as target in novel therapies for bone disorders. Research on lncRNA-based therapy represents an emerging field in bone disorders, however, some challenges has to be considered, as detailed by Meng et al. The length of lncRNAs, the difficulty of lncRNAs delivery to the bony forming surface *in vivo* and the little homology of lncRNAs among different species have limited functional *in vitro and in vivo* studies and, consequently, any translational approach. Approaches like *in vivo* injection into the mouse femoral medullary cavity or the use of hydrogel to control the local *in vivo* delivery of lncRNAs have improved this aspect. Moreover, synthesized or modified lncRNAs have been demonstrated to regulate bone cell activity similarly to the full length sequence, in *in vitro* and *in vivo* studies. Therefore, the manipulation of full length lncRNAs may overcame the homology issue, opening the possibility of their application as therapeutic drug for osteoporosis and other bone diseases.

Besides the alterations in lncRNA expression, also lncRNAs sequence mutations may have implication in bone pathophysiology. A study by Liu et al., provides insight into the role of MIR31HG polymorphisms into the pathogenesis of alcoholic osteonecrosis of the femoral head (ONFH), a common hip illness characterized by impaired microvascular circulation leading to the death of bone cells. MIR31HG genotyping in the Chinese Han male population has revealed genetic variants associated with alcoholic ONFH predisposition while rs10965059 and rs10965064 having protective effects on the occurrence of this disease, reducing ONFH risk.

The main findings on molecular mechanism of action and role as potential clinical indicators of lncRNAs are related to bone cancers onset and progression. Based on their pro- or anti-tumorigenic effects, lncRNAs can be classified as oncogenic (NEAT1, HOTAIR, ANRIL) and tumour suppressor (MALAT1, XIST, GAS5, CTD903, TUSC7). As detailed by Maroni et al., lncRNAs are emerging molecules in mediating the formation of bone metastases from primary prostate, breast and lung cancers. lncRNAs have been reported to promote cell migration, bone invasion and metastasis development by inducing epithelial-mesenchymal transition, by acting on cell adhesion molecules and metalloproteases, and by modulating cell cycle and apoptotic pathways.

The identification of lncRNAs relevant networks in the contest of bone metabolism and homeostasis, other than providing insights into the complexity of bone biology, may support the clinical implementation of these molecules as potential diagnostic and prognostic biomarkers and as targets for novel therapeutics. However, the path towards their clinical use is still long. Indeed, if from one hand, there is the dramatic need to adequately describe the role of the lncRNAs network in bone disorders, on the other hand, methodological studies should be performed in order to solve all the technical issues related to their measurement.

## Author contributions

MF: Conceptualization, Writing – original draft. MG: Writing – review & editing. CY: Writing – review & editing. GL: Writing – review & editing.
